# Supplementary material for: Effects of the Italian Law on Patient Safety and Health Professional Responsibilities Five Years after Its Approval by the Italian Parliament
Source: Healthcare (Basel). 2023 Jun 26;11(13):1858. doi: 10.3390/healthcare11131858 (PMC10340613; doi:10.3390/healthcare11131858)
Supplement: Supplementary file 1 [file healthcare-11-01858-s001.zip › healthcare-2378545-SI.pdf]

# Supplementary Material

# File S1

# Questionnaire Addressed To The Directors-Coordinators Of Regional Centers For Health Risk Management And Patient Safety

*Candido, G.; Cascini, F.; Lachman, P.; La Regina, M.; Parretti, C.; Valentini, V.; Tartaglia, R.*
*Effects of the Italian law on patient safety and health professional responsibilities, five years after its approval by Italian Parliament.*

**General instructions**

Participation is free and completely voluntary. The time required to complete the questionnaire is approximately 15 minutes. There is no grant for completion. Once completed, the questionnaire must be sent through the Google® Forms Platform.

**INFORMED CONSENT**

"Pursuant to the legislation relating to the protection of personal data (EU Regulation 2016/679 - *General Data Protection Regulation* - GDPR - applied from 25 May 2018 and in compliance with Legislative Decree 30 June 2003 n. 196 *Code regarding the protection of personal data*), the transmitted data will be collected and stored in an appropriate manner, analyzed in an anonymous and aggregated form and will be used exclusively for scientific research purposes". The research results may be published in aggregate form, therefore the identity of the participants will remain anonymous.

By continuing, you confirm your willingness to participate in the Survey and give your consent to the processing of the data provided. *

☐ **I agree**. (* Required field).

***Section 1 – Organization (9 items)***

1. Indicate the year of establishment of the Regional Center: _______.

2. How many people are on the full-time staff of the Center? Indicate the number: _____ .

*Then indicate for each qualification the NUMBER of full-time employees:*

*2.1 Physicians:____ ;*

*2.2 Nurses: _____ ;*

*2.3 Jurists-Economists: _____ ;*

*2.4 Sociologists: _____ ;*

*2.5 Psychologists: _____;*

*2.6 Trainee or postgraduate students: _____;*

*2.7 Engineers: _____;*

*2.8 Other professional figures: _____ .*

3. The coordinator of the center is:

☐ Specialist in hygiene or forensic medicine;

☐ Physician of another specialty but with training on clinical risk management and at least three years of experience;

☐ Employee with another degree but who has undergone specific training on health risk (master's, advanced course) and has at least three years' experience;

☐ Other manager without specific training on clinical risk management.

4. How often does the coordinator of the center meet with the director general of regional health to report on the work done?

☐ Every week;

☐ Every month;

☐ Less than 4 times a year;

☐ At least 4 times a year.

5. Estimate as a percentage of the total time dedicated to the following activities carried out annually by the centre:
*(The sum of all activities as a percentage must equal to 100)*

| | No dedicated time | 10% | 20% | 30% | 40% | 50% | 60% | 70% |
|---|---|---|---|---|---|---|---|---|
| *(a) Activities with citizens' associations* | ☐ | ☐ | ☐ | ☐ | ☐ | ☐ | ☐ | ☐ |
| *(b) Sentinel event analysis* | ☐ | ☐ | ☐ | ☐ | ☐ | ☐ | ☐ | ☐ |
| *(c) Information systems management (claims, sentinel events, PNE);* | ☐ | ☐ | ☐ | ☐ | ☐ | ☐ | ☐ | ☐ |
| *(d) Training* | ☐ | ☐ | ☐ | ☐ | ☐ | ☐ | ☐ | ☐ |
| *(e) Promotion and implementation of good practices, recommendations, guidelines in healthcare facilities* | ☐ | ☐ | ☐ | ☐ | ☐ | ☐ | ☐ | ☐ |
| *(f) Appropriateness analysis of treatments* | ☐ | ☐ | ☐ | ☐ | ☐ | ☐ | ☐ | ☐ |
| *(g) Proactive analysis of clinical pathways;* | ☐ | ☐ | ☐ | ☐ | ☐ | ☐ | ☐ | ☐ |
| *(h) Research activity* | ☐ | ☐ | ☐ | ☐ | ☐ | ☐ | ☐ | ☐ |

6. While Law 24/2017 does not provide any financial commitment for the Regional Centres, what funding has been requested/obtained by your center for carrying out training and research initiatives or for acquiring staff? *(You can enter multiple answers)*

☐ Funding from regional projects;

☐ Funding from national projects (National Agency for Regional Health Services, Health Ministry, others);

☐ Funding from European projects;

☐ None;

☐ Other: ______________________________________________ .

7. How often does the Center meet with the peripheral clinical risk managers:

☐ Every week;

☐ Every month;

☐ Less than 4 times a year;

☐ At least 4 times a year.

8. Does the Center have constant collaborations with scientific societies and professional associations?

☐ Yes;

☐ No.

9. Which of the following indicators does the Center use to monitor the safety of care? *
*(You can enter multiple answers)*

☐ OCse indicators of patient safety;

☐ PNE* indicators;

☐ Claims;

☐ Sentinel events;

☐ Nobody;

☐ Other: ______________________________________________________________ .

**PNE (Programma Nazionale Esiti): National Outcome program calculates the mortality for some medical, surgical and other invasive procedures in Italian hospitals.*

*Section 2 – Essential Activities (11 items)*

10. Does the Center establish actions to be taken each year to facilitate risk management and increase safety?

☐ Yes;

☐ No.

*10.1 If the answer is yes, please indicate at least the three most important actions ones established for 2022:* ______________________________________________
______________________________________________ .

11. Is the manager of the center or one of his collaborators a permanent member of the claims evaluation committee?

☐ Yes;

☐ No.

12. Has the Center promoted the updating or implementation of recommendations, good practices, guidelines for the safety of care in 2022?

☐ Yes;

☐ No.

*12.1 If the answer is yes, please indicate which and how (training, information campaigns, processing/review of procedures, etc.):*______________________________
______________________________________________ .

13. Has the Center promoted synergies and collaborations between peripheral CRMs, in order to favor a homogeneous application of national and regional indications?

☐ Yes;

☐ No.

*12.1 If the answer is yes, with actions referring to which activities?* ____________
______________________________________________ .

14. Does the Center publish a report on the safety of care with data on litigation and sentinel events annually on the regional website?

☐ Yes;

☐ No.

15. Has the Center promoted the implementation of the adverse events reporting and learning system in all the regional healthcare facilities?

☐ Yes;

☐ No.

16. Does the Center participate directly in the analysis of sentinel events?

☐ Yes;

☐ No.

17. From whom does the Center receive formal feedback on the prevention measures adopted? *(You can enter multiple answers)*

☐ Formal response from the Ministry;

☐ Formal response from Age.na.s.;

☐ Formal response from Central Institutions;

☐ No response;

☐ Other: ________________________________________________ .

18. Is the Center constantly updated on the best practices developed and adopted by a healthcare facility and does it disseminate them to all the others?

☐ Yes;

☐ No.

19. Does the Center carry out annual visits to healthcare facilities to verify the correct implementation of good practices (Regional patient safety walkarounds)?

☐ Yes;

☐ No.

20. Are the annual reports of peripheral healthcare companies on the safety of care subject to an evaluation and feedback by the Regional Center?

☐ Yes;

☐ No.

***Section 3 - Monitoring of health care companies (1 item)***

21. The Center annually monitors the healthcare companies through:
*(You can enter multiple answers)*

☐ OCSE/OECD patient safety indicators;

☐ PNE data;

☐ Claims data;

☐ Data on sentinel events;

☐ None;

☐ Other: ______________________________ .

*21.1 PNE data - If the answer is Yes, please indicate the audits carried out in the event of anomalous data compared to the mean national outcomes in 2022:* ______________________________ .

*21.2 Data on sentinel events - If the answer is Yes, please indicate the number of audits carried out in 2022:* ______________________________ .

***Section 4 - Communication (7 items)***

22. Does the Center promote public communication activities on patient safety and quality of care?

☐ Yes;

☐ No.

*22.1 If the answer is Yes, please indicate which activities carried out in the last two years:* ___
________________________________________ .

23. Does the Center carry out training or other initiatives on the theme of *"difficult communication"* (disclosure)?

☐ Yes;

☐ No.

24. Does the Center have a web page by which it communicates its activity to citizens?

☐ Yes;

☐ No.

25. Does the Center have a private site by which it communicates the activities carried out and share the materials produced with the peripheral CRMs?

☐ Yes;

☐ No.

26. Has the Center organized and promoted good practice campaigns to raise awareness among professionals?

☐ Yes;

☐ No.

27. Has the Center organized and promoted campaigns to make citizens aware of the issue of security?

☐ Yes;

☐ No.

28. In the last two years, the Center has published its works in scientific journals with IF?

☐ Yes;

☐ No.

*28.1 If the answer is Yes, please indicate which:* ______________________________________
______________________________________________________________________ .

***Section 5 - Training (2 items)***

29. Does the Center periodically promote refresher/training courses for peripheral CRMs in collaboration with the University?

☐ Yes;

☐ No.

30. In the last 24 months, the Center has organized training activities (courses, seminars, meetings, etc.) for:
*(You can enter multiple answers)*

☐ Healthcare workers (physicians, nurses, etc.);

☐ Facility top management;

☐ Other professional figures;

☐ None.

*Section 6 - COVID-19 pandemic management (3 items)*

31. Was the Director of the Center involved in the management of the first phase of the pandemic?

☐ Yes;

☐ No.

32. Was the Director of the Center one of the members of the regional task force during the pandemic?

☐ Yes;

☐ No.

33. During the pandemic, did the Center perform a support function for front-line operators with respect to the measures to be taken for patient safety (Recommendations developed by the *Istituto Superiore di Sanità* - ISS, the *International Society for Quality in Health Care* - ISQua, and other institutional bodies)?

☐ Yes;

☐ No.

# Supplementary Material

# File S2

# Questionnaire Addressed To Facilities Clinical Risk Managers (CRMs)

*Candido, G.; Cascini, F.; Lachman, P.; La Regina, M.; Parretti, C.; Valentini, V.; Tartaglia, R.*
*Effects of the Italian law on patient safety and health professional responsibilities,*
*five years after its approval by Italian Parliament.*

**General instructions**

Participation in the study consists in completing a questionnaire on the most important activities carried out by the Clinical Risk Managers. Participation is anonymous, free and completely voluntary. The time required to complete the questionnaire is approximately 15 minutes. There is no grant completion. Once completed, the questionnaire must be sent through the Google® Forms Platform.

**INFORMED CONSENT**

"Pursuant to the legislation relating to the protection of personal data (EU Regulation 2016/679 - *General Data Protection Regulation* - GDPR - applied from 25 May 2018 and in compliance with Legislative Decree 30 June 2003 n. 196 *Code regarding the protection of personal data*), the transmitted data will be collected and stored in an appropriate manner, analyzed in an anonymous and aggregated form and will be used exclusively for scientific research purposes". The research results may be published in aggregate form, therefore the identity of the participants will remain anonymous.

By continuing, you confirm your willingness to participate in the Survey and give your consent to the processing of the data provided. *

☐ **I agree**. (* Required field).

*Section 1 - General and organizational characteristics (8 Items)*

1. Year of birth: _______.

2. Gender:

☐ Male;

☐ Female.

3. Workplace region:

☐ Abruzzo;

☐ Basilicata;

☐ Calabria;

☐ Campania;

☐ Emilia-Romagna;

☐ Friuli Venezia Giulia;

☐ Lazio;

☐ Liguria;

☐ Lombardy;

☐ Marche;

☐ Molise;

☐ Piedmont;

☐ Puglia;

☐ Sardinia;

☐ Sicily;

☐ Tuscany;

- ☐ Umbria;
- ☐ Valle D'Aosta;
- ☐ Veneto;
- ☐ Autonomous Province of Bolzano;
- ☐ Autonomous Province of Trento.

4. Workplace:

- ☐ Hospital and University Hospital;
- ☐ Local Health Company;
- ☐ IRCCS (Istituti di Ricovero e Cura a Carattere Scientifico): Institutes for Hospitalization and Treatment of a Scientific Nature.
- ☐ Accredited Private Facility;
- ☐ Other.

5. Professional role:

- ☐ Chief of a “complex” unit;
- ☐ Chief of a “simple” unit;
- ☐ Medical Director;
- ☐ Health Professions Director;
- ☐ Non-Healthcare Manager.

6. How many years have you been working as a company clinical risk manager?
*Indicate the number:* _______.

7. Qualification of the CRM at the facility where you work:

☐ Specialist in hygiene or forensic medicine;

☐ Physician of another specialty but with training on clinical risk management and at least three years of experience;

☐ Employee with another degree but who has undergone specific training on health risk (master's, advanced course) and has at least three years' experience;

☐ Other manager without specific training on clinical risk management.

8. How many full-time professionals does your staff consist of? *Indicate the number: _____;*

*Then indicate for each qualification the NUMBER of full-time employees:*

*8.1 Physicians:____ ;*

*8.2 Nurses: _____ ;*

*8.3 Jurists-Economists: _____ ;*

*8.4 Sociologists: _____ ;*

*8.5 Psychologists: _____;*

*8.6 Trainee or postgraduate students: _____;*

*8.7 Engineers: _____;*

*8.8 Other professional figures: _____ .*

*Section 2 - Activities and tools (11 Items)*

9. During the last 36 months how many audits have you carried out?
*Indicate the number:* _____ .

10. Following the PNE* data, have any clinical audits been carried out in the last 36 months?

☐ Yes;

☐ No.

**PNE (Programma Nazionale Esiti): National Outcome program calculates the mortality for some medical, surgical and other invasive procedures in all Italian hospitals.*

11. How many incident reportings have you collected in the last 36 months for adverse events (including near misses)? *Indicate the number:* _____ .

12. How many proactive analyzes have you conducted in the last 36 months?
*Indicate the number:* _____ .

13. Which of the following indicators do you use to monitor the safety of care in your healthcare company? *(You can enter multiple answers)*

☐ OCse indicators of patient safety;

☐ PNE indicators;

☐ Claims;

☐ Sentinel Events;

☐ Adverse events;

☐ Incident reporting;

☐ Near misses;

☐ Other.

14. Do you carry out analyzes to evaluate the risk of inappropriateness?

☐ Yes;

☐ No.

*14.1 If the answer is Yes, please indicate which indicators you use:* ____________________
__________________________________________________________________________ .

15. How many training courses on clinical risk management have you organized in the last 36 months for employees? *Indicate the number:* _____ .

16. Does your unit have a dedicated page on the company website to publish the annual report and useful information for citizens?

☐ Yes;

☐ No.

17. How many events (meetings, conferences, seminars, study days) have you held over the last 36 months on clinical risks for the public? *Indicate the number:* ____.

18. How many awareness campaigns on patient safety aimed at healthcare professionals have you carried out in the last 36 months? *Indicate the number:* _____ ;

*18.1 Pleace specify which awareness campaigns you have carried out in the last 36 months:*
__________________________________________________________________________
__________________________________________________________________________ .

19. Have you ever been asked to hand over minutes or other documents related to audits to the judiciary after Law 24/2017 was passed?

☐ Yes;

☐ No.

***Section 3 - Relationship with the Law n. 24/2017 (3 Items)***

20. What barriers did you encounter in carrying out your role?
*(You can enter multiple answers)*

☐ Lack of knowledge and awareness of its positive impact;

☐ Lack of dedicated economic resources;

☐ Lack of dedicated human resources;

☐ Bureaucracy and hierarchies that prevent your activity;

☐ Resistance to change;

☐ Lack of leadership oriented towards safety and quality;

☐ Other.

21. What change do you think have been made by the Gelli/Bianco Law?
*(You can enter multiple answers)*

☐ Better patient safety;

☐ Greater protection of healthcare workers;

☐ Greater clarity in accountability;

☐ Re-modulation of the costs of insurance policies;

☐ Litigation prevention;

☐ Other: ________________________________________________ .

22. What limits do you think characterize the Gelli/Bianco Law?

____________________________________________________________

____________________________________________________________ .

***Section 4 - Training (2 Items)***

23. What training/re-training courses have you attended on the topic of clinical risk management in the last 36 months? *(You can enter multiple answers)*

☐ Extra company re-training course;

☐ Company re-training course;

☐ Specific Masters;

☐ Online course;

☐ None;

☐ Other: ________________________________________________ .

24. How many papers on journals with impact factor have been published in the last 36 months by your unit? *Indicate the number:* _____ ;

*24.1 Please, indicate the topic:* ____________________________________
____________________________________________________ .

*Section 5 - Interaction with Regional Centers (2 Items)*

25. In the last 36 months, how frequently have you had meetings with the Regional Center?

- ☐ Weekly;
- ☐ Monthly;
- ☐ Annual;
- ☐ Occasional;
- ☐ None.

26. For what reasons did the Regional Center promote these meetings?
*(You can enter multiple answers)*

- ☐ Audit for sentinel events;
- ☐ Benchmarking among the clinical risk management units of the region;
- ☐ Standardization of procedures and best practices;
- ☐ Sharing of guidelines and elaboration of a regional plan for the safety of care;
- ☐ Other: ____________________________________________ .

*Section 6 - Management of the COVID-19 Pandemic (3 Items)*

27. Were you involved in the management of the first phase of the pandemic?

☐ Yes;

☐ No.

28. Were you one of the members of the company task force during the pandemic?

☐ Yes;

☐ No.

29. During the pandemic, did you perform a support function for front-line operators with respect to the measures to be taken for patient safety (Recommendations developed by the *Istituto Superiore di Sanità* - ISS, the *International Society for Quality in Health Care* - ISQua, and other institutional bodies)?

☐ Yes;

☐ No.
